# Attention-Enhanced ResNet–U–Net for Automated Colorectal Tumor Segmentation in CT Scans

**DOI:** 10.3390/life16081309

**Published:** 2026-08-10

**Authors:** Lucian Mihai Florescu, Cosmin Vasile Obleagă, Mădălin Mămuleanu, Ioana Andreea Cîrlig, Alesandra Florescu, Mihai Alexandru Ene, Aurelia Ștefania Domenco, Alexandru Marian Olaru, Alexandra Gabriela Cosmina Țâru, Raluca Elena Nica, Rossy Vlăduț Teică, Ioana Andreea Gheonea

**Affiliations:** 1 Department of Radiology and Medical Imaging, University of Medicine and Pharmacy of Craiova, 200349 Craiova, Romania; lucian.florescu@umfcv.ro (L.M.F.); raluca.nica@umfcv.ro (R.E.N.); rossy.teica@umfcv.ro (R.V.T.); ioana.gheonea@umfcv.ro (I.A.G.); 2 Department of Surgical Oncology, University of Medicine and Pharmacy of Craiova, 200349 Craiova, Romania; 3 Department of Automatic Control and Electronics, University of Craiova, 200585 Craiova, Romania; 4 Doctoral School, University of Medicine and Pharmacy of Craiova, 200349 Craiova, Romania; andreea.cirlig97@gmail.com (I.A.C.); mihai.ene@umfcv.ro (M.A.E.); domenco.aura@gmail.com (A.Ș.D.); 5 Department of Rheumatology, University of Medicine and Pharmacy of Craiova, 200349 Craiova, Romania; alesandra.florescu@umfcv.ro; 6 Department of Radiology and Medical Imaging, Emergency Clinical County Hospital of Craiova, 200642 Craiova, Romania; alex.olaru89@yahoo.com (A.M.O.); alexandra.taru@yahoo.com (A.G.C.Ț.)

**Keywords:** colon cancer, deep learning, automated tumor segmentation, attention-enhanced U-Net, computed tomography

## Abstract

Automated colorectal tumor segmentation on routine computed tomography (CT) remains technically challenging because of limited soft-tissue contrast, heterogeneous tumor morphology, complex bowel anatomy, and marked imbalance between tumor and background pixels. This retrospective technical feasibility study aimed to develop and evaluate an attention-enhanced ResNet50–U-Net architecture for automated segmentation of primary colorectal tumors on contrast-enhanced abdominopelvic CT images. The dataset comprised 493 axial CT image–mask pairs containing visible colorectal tumors, including 70 institutional images obtained from 25 patients and 423 images from publicly available colorectal cancer imaging resources. Reference masks were generated by manual tumor delineation performed by an experienced radiologist and reviewed by a second abdominal radiologist, with uncertain contours resolved by consensus. The proposed model combined an ImageNet-pretrained ResNet50 encoder with an attention-guided U-Net decoder and was trained using a composite Binary Cross-Entropy and Focal Tversky loss function to address foreground–background class imbalance. Performance was assessed using five-fold image-level cross-validation. Predicted probability maps were binarized using a threshold of 0.5, and segmentation metrics were calculated through global micro-averaging within each validation fold. The model achieved a mean Intersection over Union of 0.7406 ± 0.0276, a Dice similarity coefficient of 0.8507 ± 0.0182, a pixel-level sensitivity of 0.9559 ± 0.0266, and a pixel-level background specificity of 0.9956 ± 0.0003. Qualitative assessment demonstrated substantial spatial agreement between predicted masks and expert annotations, although minor boundary discrepancies and small false-positive components were observed. These findings support the technical feasibility of attention-enhanced encoder–decoder architectures for colorectal tumor segmentation on routine CT images. However, because validation was performed at the image level, entirely tumor-negative images were not included (preventing the evaluation of clinical specificity and false-positive rates), and no independent external test cohort was available, further patient-level, multicenter, and DICOM-based validation is required before clinical implementation. While the model demonstrated robust internal performance, future studies must prioritize large-scale external validation and patient-level cross-validation to rigorously assess generalizability and specificity.

## 1. Introduction

According to the most recent data from the Global Cancer Observatory, colorectal cancer was diagnosed in more than 1.9 million individuals in 2022, making it the third most common malignancy worldwide. Colorectal cancer also remains one of the leading causes of cancer-related mortality, with more than 900,000 deaths reported in 2022 [1,2]. These figures underline the persistent global burden of the disease and highlight the need for effective screening strategies, earlier diagnosis, accurate staging, and optimized treatment planning [2].

The incidence and mortality of colorectal cancer show marked geographic variability, reflecting complex interactions between dietary patterns, cultural factors, socioeconomic development, access to healthcare, and the implementation of screening programs. Asia and Europe account for a substantial proportion of global colorectal cancer cases and deaths. In Asia, the incidence of colorectal cancer has increased steadily and, in several East and Southeast Asian countries, is approaching rates historically observed in Western populations. In 2022, Asia accounted for approximately 50.2% of global colorectal cancer cases, with 966,400 new cases and 462,300 deaths reported [2,3]. Countries such as Japan show particularly high incidence rates, whereas countries with a lower Human Development Index may report lower incidence but are projected to experience increasing mortality by 2050 [3,4]. These trends are largely associated with population aging, urbanization, westernized dietary habits, obesity, smoking, physical inactivity, and other lifestyle-related risk factors [5,6]. In parallel with imaging-based approaches, recent biomarker studies have investigated neuropeptide-related pathways in colorectal adenocarcinoma, including calcitonin gene-related peptide, calcitonin receptor-like receptor, substance P, and their receptors, highlighting the increasing interest in multimodal characterization of colorectal cancer [7,8].

In this context, artificial intelligence (AI) and deep learning methods have gained increasing relevance in medical image analysis. The field has evolved from traditional semi-automated computer-aided diagnosis systems based on handcrafted features, such as intensity thresholding, region growing, and active contour models, toward end-to-end deep learning frameworks capable of learning complex imaging patterns directly from data [9]. Traditional segmentation approaches are often insufficient for colorectal tumors because of their heterogeneous morphology, irregular borders, and limited contrast against adjacent soft tissues [9]. Convolutional neural networks (CNNs) have addressed many of these limitations by enabling hierarchical feature extraction from raw image data, thereby reducing the dependence on subjective manual feature engineering [10].

Among CNN-based segmentation methods, the U-Net architecture has become one of the most influential frameworks in biomedical image segmentation [11]. Its symmetric encoder–decoder structure and skip connections allow the integration of high-resolution spatial information from the contracting path with semantic information from the expanding path [11]. This design enables simultaneous object localization and pixel-level delineation, which is particularly important for medical segmentation tasks [12]. The anatomical complexity of the colon, including its tortuous tubular configuration, variable luminal content, and proximity to multiple abdominal and pelvic structures, makes automated colorectal tumor segmentation especially challenging [9]. Nevertheless, modern deep learning models have shown substantial potential across a wide range of medical image segmentation tasks, outperforming earlier approaches in several organ systems and imaging applications [13].

As the field has matured, the focus has shifted from basic encoder–decoder architectures toward enhanced feature extraction, attention mechanisms, and model designs that are better adapted to the specific challenges of CT imaging. Accurate segmentation of colorectal tumors may support quantitative tumor assessment, treatment planning, response evaluation, and the development of downstream radiomics or prognostic models [14]. However, segmentation on routine abdominal CT remains technically demanding because tumor visibility depends on lesion size, bowel distension, luminal content, contrast enhancement, pericolic fat planes, and image acquisition variability. Small or subtle tumors may be obscured by fecal material, collapsed bowel loops, or the absence of secondary signs such as pericolic fat stranding [9].

Attention mechanisms are particularly relevant for colorectal tumor segmentation because they may suppress irrelevant activations from adjacent bowel loops, vessels, mesenteric fat, and pelvic structures while preserving tumor-related features. In parallel, residual encoder backbones such as ResNet can improve feature extraction by facilitating gradient propagation and enabling the use of deeper representations. Combining a ResNet-based encoder with an attention-guided U-Net decoder may therefore provide a suitable architecture for colorectal tumor segmentation on CT images.

The present study aims to develop and evaluate an attention-enhanced ResNet-U-Net architecture for automated colorectal tumor segmentation on contrast-enhanced abdomino-pelvic CT images. The proposed approach is intended as a technical step toward computer-assisted tumor delineation in routine CT imaging, rather than as a replacement for colonoscopy, histopathological diagnosis, or expert radiological interpretation. By improving automated segmentation of colorectal tumor regions, this model may contribute to future AI-assisted workflows for lesion quantification, radiological decision support, and treatment planning.

## 2. Materials and Methods

### 2.1. Study Design and Ethical Approval

This retrospective imaging study was conducted in accordance with the principles of the Declaration of Helsinki and was approved by the Committee of Ethics and Academic and Scientific Deontology of the University of Medicine and Pharmacy of Craiova, Romania, approval number 434/12 December 2025. All imaging data were fully anonymized before analysis. Because of the retrospective design and the use of anonymized imaging data, the requirement for individual informed consent was waived by the ethics committee.

### 2.2. Image Dataset and Case Selection

The final dataset consisted of 493 axial contrast-enhanced abdominopelvic CT images containing visible primary colorectal tumors. Of these, 70 images were retrospectively obtained from 25 patients examined at the Department of Radiology and Medical Imaging, University of Medicine and Pharmacy of Craiova, Romania, whereas 423 images were obtained from publicly available colorectal cancer imaging resources, including the StageII-Colorectal-CT dataset from The Cancer Imaging Archive [15]. No entirely tumor-negative CT images with empty reference masks were included in the final dataset.

The institutional cohort was retrospectively identified from patients who underwent portal venous phase contrast-enhanced abdominopelvic CT at our institution between January 2022 and December 2025 using a Siemens Biograph mCT 20-slice scanner (Siemens Healthcare GmbH, Erlangen, Germany) and had a documented primary colorectal malignancy. Patient-level inclusion criteria were: (a) availability of a portal venous phase contrast-enhanced CT examination; (b) presence of a visible primary colorectal tumor; (c) adequate image quality for reliable tumor delineation; and (d) histopathological confirmation of colorectal cancer. Patient-level exclusion criteria were: (a) absence of intravenous contrast administration; (b) absence of an identifiable primary colorectal tumor on CT; (c) severe motion or metallic artifacts limiting tumor assessment; (d) postoperative or post-treatment examinations without a clearly delineable residual primary tumor; (e) incomplete imaging data; and (f) duplicate examinations. After patient-level eligibility had been established, axial slices containing a clearly visible and reliably delineable primary colorectal tumor were selected for manual annotation, resulting in 70 images from 25 patients.

Publicly available images were included only when the presence of colorectal cancer was clearly documented and when the tumor was visible on axial contrast-enhanced CT images.

The inclusion criteria for tumor-positive images were:

(a) Axial contrast-enhanced abdomino-pelvic CT images;

(b) Visible primary colorectal tumor on the selected slice;

(c) Histopathological confirmation of colorectal cancer when available;

(d) Adequate image quality for tumor delineation;

(e) Availability of an image suitable for manual annotation and binary mask generation.

The exclusion criteria for tumor-positive images were:

(a) Non-contrast CT images;

(b) Severe motion artifacts;

(c) Severe metallic artifacts impairing image interpretation;

(d) Duplicate images;

(e) Images with insufficient quality for reliable analysis.

Because the final dataset consisted of anonymized exported image files, complete patient-level metadata could not be retrieved for all public-source cases. Therefore, the present study should be interpreted as an image-level technical feasibility study of colorectal tumor segmentation rather than as a definitive patient-level clinical validation.

### 2.3. Image Export and Preprocessing

All CT images were exported in portable network graphics format. Before model training, images were visually reviewed to confirm tumor visibility and adequate image quality. The images were resized to 512 by 512 pixels to ensure a uniform input dimension for the neural network. To ensure compatibility with the ImageNet-pretrained ResNet50 encoder, the single grayscale channel was replicated three times to generate a three-channel input compatible with the ImageNet-pretrained ResNet50 encoder. Pixel values were first scaled from [0, 255] to [0, 1]. The resulting values were then channel-wise standardized using the ImageNet mean and standard deviation.

For internally retrieved CT examinations, images were exported using a standardized abdominal soft-tissue window setting preset. For public-source images, the available exported image appearance was preserved because original DICOM windowing parameters were not consistently available.

Because the dataset was based on exported PNG images rather than original DICOM volumes, voxel-level Hounsfield Unit information and full volumetric metadata were not available during model training. This limitation was considered when interpreting model performance.

### 2.4. Tumor Annotation and Ground-Truth Mask Generation

Manual tumor delineation was performed using QuPath v0.6.0. The visible primary colorectal tumor was manually outlined on each axial CT image, and the corresponding binary segmentation mask was exported for supervised model training. The segmentation target included the visible tumor mass on the selected axial slice. Adjacent lymph nodes, pericolic inflammatory changes, vessels, bone structures, and distant metastatic lesions were excluded from the tumor mask.

**Figure 1:**
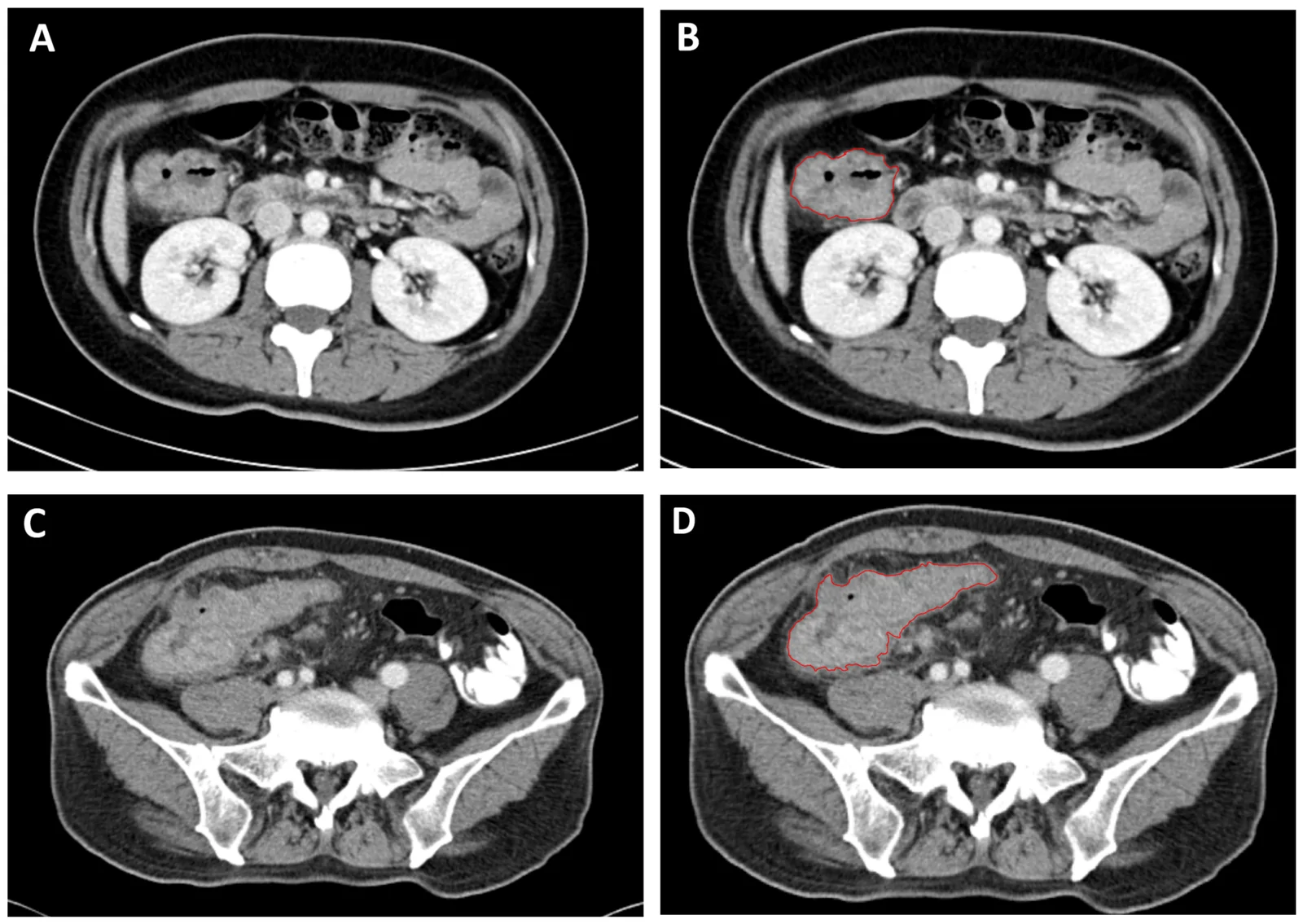
Figure 1 illustrates the differences between raw and segmented colon tumors on CT scans using QuPath. (**A**) Raw contrast-enhanced CT image of a patient with colon cancer; (**B**) segmented colon cancer CT using QuPath; (**C**) raw contrast-enhanced CT image of a patient with colon cancer; (**D**) segmented colon cancer CT using QuPath.

Tumor annotations were performed by L.M.F. with 10 years of experience and reviewed by I.A.G. with 20 years of experience in abdominal imaging. Discrepant or uncertain contours were corrected by consensus. Figure 1A–D presents several colorectal tumor segmentation images using the QuPath application.

The dataset masks were computed using QuPath scripting options. To prepare the CT data for training, the expert annotations were standardized into a unified coordinate system. This process involves mapping subjective clinical delineations into a discrete mathematical framework to facilitate supervised training. The segmentation task is modeled as a pixel-wise binary classification problem. To ensure consistency across the various acquisition protocols, we define a rigorous mathematical protocol for mask generation. By converting polygonal expert regions into discrete binary maps, we establish a definitive training target as follows:

Let I denote the spatial domain of the CT scan slice, with dimensions W×H, such that any given pixel coordinate is defined as (x,y)∈[0,W−1]×[0,H−1].

Let $A=\{a_{1},a_{2},\ldots ,a_{N}\}$ represent the discrete set of N expert-annotated Regions of Interest (ROIs) corresponding to tumor formations. Each annotation $a_{i}$ corresponds to a closed geometric shape that bounds a specific subset of the spatial domain, denoted as $S_{i}\subset I$. The objective is to compute a discrete binary segmentation mask $M\in \{0,1{\}}^{W\times H}$, mapping each pixel coordinate (x,y) to a binary class label, where **0** represents the background (healthy tissue/empty space) and **1** represents the foreground (tumor region).

The ground truth mask generation is formalized as follows:

$$M_{x,y}=\left\{\begin{array}{ll} 1, & \mathrm{if}\;(x,y)\in \overset{N}{\underset{i=1}{⋃}}\;\;S_{i}\\ 0, & \mathrm{otherwise} \end{array}\right.$$

The following procedure details the implementation of the mask generation pipeline using the QuPath object hierarchy (Algorithm 1).
**Algorithm 1** Automated Ground Truth Mask Rasterization**Input**:
$I_{server}$: The ImageServer object containing the raw CT scan metadata.A: The hierarchical list of PathObject annotations.ρ: The downsample factor (where ρ=1 denotes full resolution).**Output**:
A lossless binary PNG image $I_{mask}$ representing the rasterized mask ***M***.**Procedure**:
1: Extract dimensions: $W\leftarrow \lfloor \mathrm{width}(I_{server})/\rho \rfloor$, $H\leftarrow \lfloor \mathrm{height}(I_{server})/\rho \rfloor$
2: Initialize zero-matrix: $M\leftarrow 0_{W\times H}$ (Allocated as an 8-bit grayscale image buffer)
3: Initialize 2D graphics context: G←CreateGraphics(M)
4: Set rendering space: ***G***.SetScale(1/ρ,1/ρ)
5: ***G***.SetColor(255) ▹ Foreground tumor class intensity (White)
6: for each a∈A do
7:  R←
***a***.GetROI()
8:    if R≠∅ then 
9:    S←ExtractGeometricShape(R)
10:    ***G***.Fill(***S***) Rasterizes ***S*** into ***M***, updating all (x,y)∈S↦255
11:    end if
12: end for 
13: ***G***.Dispose()
14: Export ***M*** to standard lossless I/O stream (e.g., ‘PNG’)


### 2.5. Dataset Splitting and Cross-Validation

Model performance was evaluated using five-fold cross-validation. The 493 image-mask pairs were divided into five folds, with each fold used once as the validation subset while the remaining four folds were used for training. Because complete patient-level identifiers were not available for all anonymized public-source images, the cross-validation was performed at the image level. To ensure uniform handling across all data sources, given the lack of patient identifiers in the public datasets, the entire dataset, was pooled and randomly partitioned strictly at the image level. Consequently, images belonging to the same patient were not constrained to the same fold. This represents an important limitation, as adjacent or related slices from the same patient could not be systematically excluded from different folds. Therefore, the results should be interpreted as preliminary image-level segmentation performance rather than definitive patient-level generalization.

To prevent model overfitting and enhance generalizability, a robust data augmentation pipeline was applied exclusively to the training set using the Albumentations library. The pipeline consisted of both spatial and pixel-level transformations applied with specific execution probabilities (p). Spatial augmentations included random Horizontal and Vertical Flips (p=0.5 each). To simulate natural anatomical variations, complex geometrical distortions were applied, including Shift-Scale-Rotate (shift limit: ±10%, scale limit: ±10%, rotation limit: $\pm 30^{{}^{\circ}}$, p=0.8 with constant border padding), Elastic Transformations (α=120, σ=6.0, $\alpha_{\mathrm{affine}}=3.6$, p=0.4), and Grid Distortions (p=0.4). Finally, to ensure robustness against varying CT scanner calibrations and noise, pixel-level intensity transforms were incorporated, specifically Random Brightness and Contrast adjustments (p=0.4) and Gaussian Blur (p=0.2).

### 2.6. Network Architecture

To achieve pixel-wise segmentation of colorectal tumors on CT images, we developed a hybrid ResNet50-Attention U-Net framework. This architecture integrates the feature extraction capability of a pre-trained ResNet50 encoder with the localization capacity of an attention-guided U-Net decoder. Each single-channel grayscale CT image was duplicated across three channels (converted to RGB format) to ensure architectural and statistical compatibility with the ImageNet-pretrained ResNet50 encoder. Furthermore, prior to passing the inputs to the network, all three-channel image tensors were globally normalized using the standard ImageNet channel-wise mean (0.485, 0.456, 0.406) and standard deviation (0.229, 0.224, 0.225).

**Figure 2:**
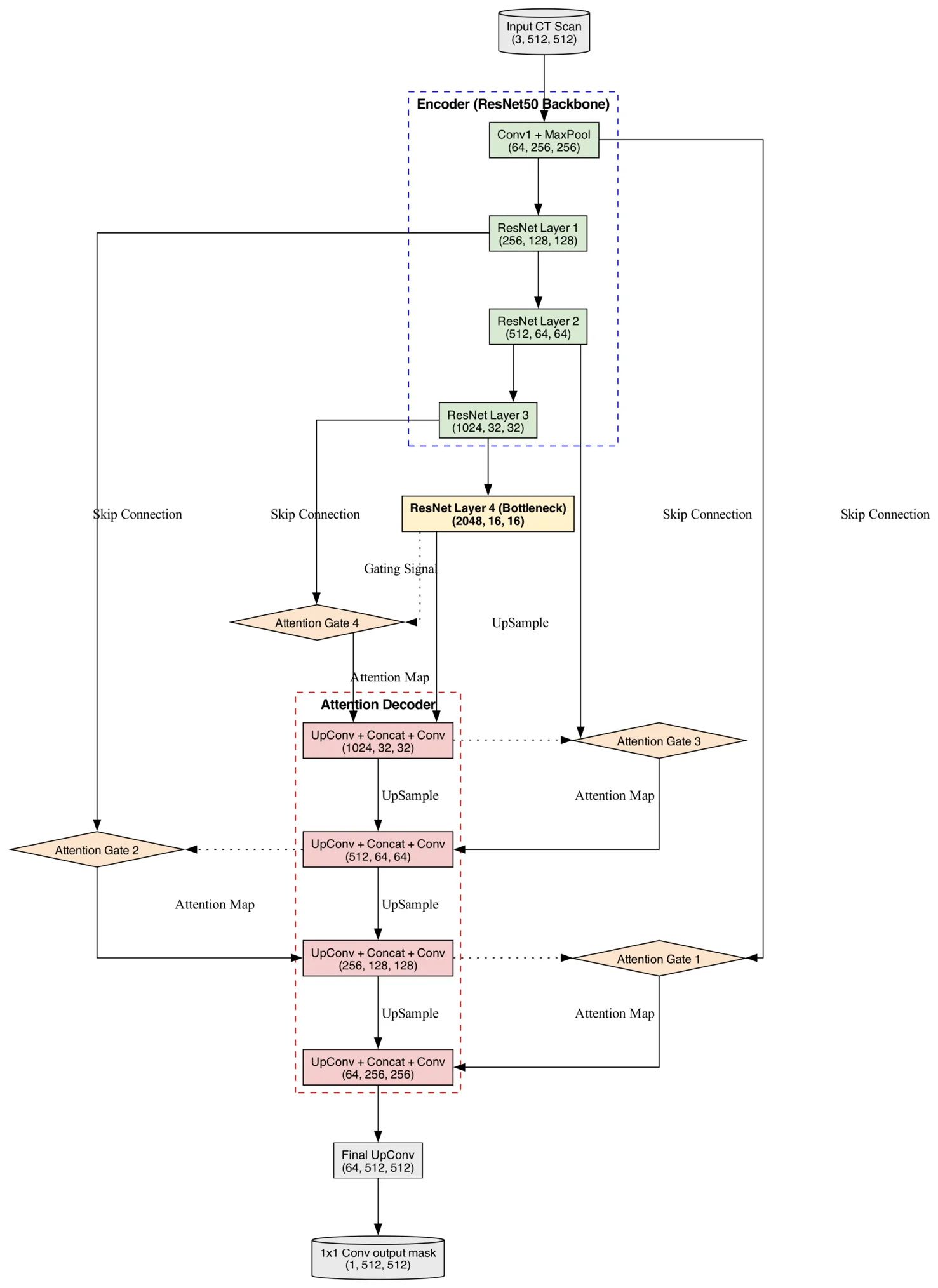
Architecture of the proposed method: ResNet50-Attention U-Net.

**Figure 3:**
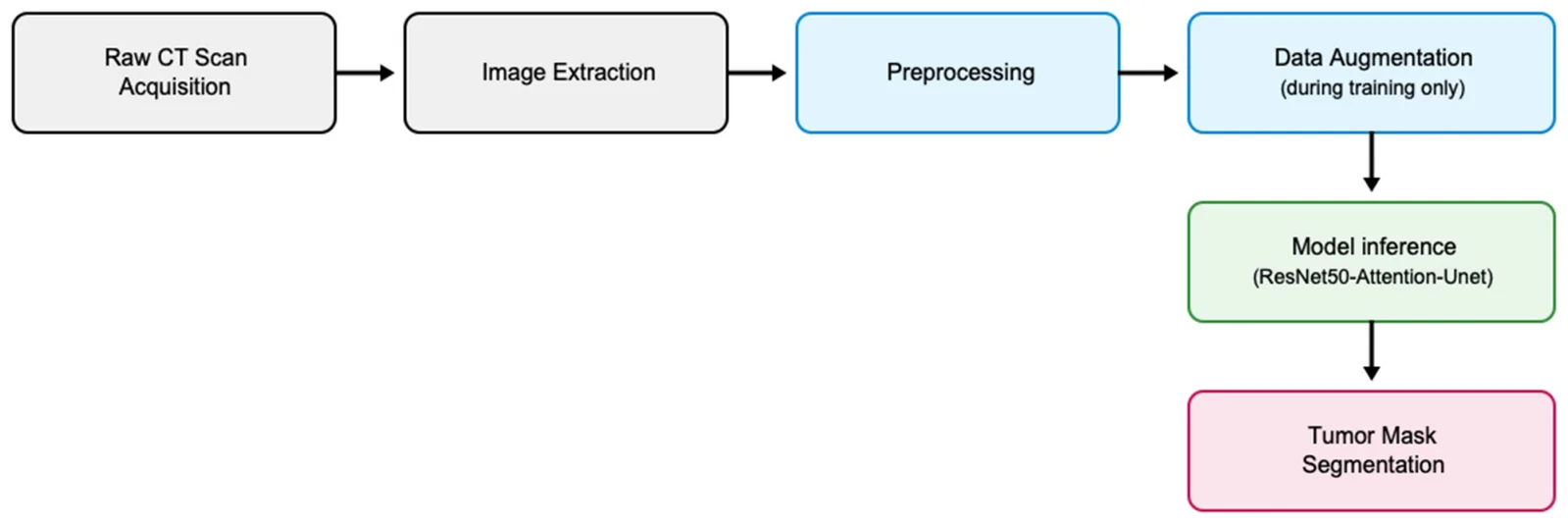
Workflow diagram illustrating the complete pipeline from CT acquisition to segmentation output.

Given the relatively limited number of tumor-positive images and the class imbalance inherent to colorectal tumor segmentation, training a deep convolutional network from scratch was considered at increased risk of overfitting. The diagram of the proposed method is presented in Figure 2 while the entire flow of the proposed method is presented in Figure 3.

### 2.7. Attention-Guided Decoder

In colorectal cancer imaging, lesions exhibit considerable variability in size, shape, and contrast when compared to adjacent healthy tissue. Conventional U-Net architectures typically concatenate encoder feature maps directly with decoder feature maps, which frequently results in the inclusion of redundant background information, such as surrounding organs or bone. To mitigate the influence of irrelevant background features and emphasize significant foreground regions, we incorporated Attention Gates (AGs) [16] into the skip connections. The Attention Gate assigns a scalar attention coefficient to each pixel.

Utilizing the deeper, coarser gating signal and the higher-resolution feature map from the encoder, the attention mechanism is defined as follows:

$$q_{att}=\psi^{T}\left(\sigma_{1}\left(W_{g}^{T}g+W_{x}^{T}x_{l}+b_{g}\right)\right)+b_{\psi }$$

$$\alpha =\sigma_{2}(q_{att})$$

$${\hat{x}}_{l}=\alpha \cdot x_{l}$$

where $\sigma_{1}$ is a ReLU activation function, $\sigma_{2}$ is a Sigmoid activation function, and $W_{x},W_{g},\psi$ are trainable linear transformations (1 × 1 Convolutions) mapping to an intermediate dimensional space. The resulting attention weights α are element-wise multiplied with the skip-connection $x_{l}$ to produce the filtered feature map ${\hat{x}}_{l}$, successfully forcing the network to structurally focus on cancerous tissue representations rather than surrounding noise.

### 2.8. Loss Function: Binary Cross-Entropy Combined with Focal Tversky Loss

Medical image segmentation naturally severely suffers from class imbalance; tumorous pixels normally occupy less than 5% of the total cross-sectional CT slice area, meaning the network is biased to heavily favor predicting background pixels. To counteract this, we propose a composite loss function containing Binary Cross-Entropy (BCE) and Focal Tversky Loss (FTL). Binary Cross-Entropy (BCE) loss provides smooth and consistent pixel-level gradients necessary for stable initial convergence. It is defined as

$$L_{BCE}=-\frac{1}{N}\sum_{i=1}^{N}\;\left[y_{i}log(p_{i})+(1-y_{i})log(1-p_{i})\right]$$

To specifically address severe class imbalance and highly penalize False Negatives (FN)—a critical necessity in oncology where missing a tumor is far more detrimental than a false positive—we incorporate the Focal Tversky Loss [17]. The Tversky Index (TI) is generalized as

$$TI_{c}=\frac{\sum_{i=1}^{N}\;\;p_{ic}y_{ic}}{\sum_{i=1}^{N}\;\;p_{ic}y_{ic}+\alpha \sum_{i=1}^{N}\;\;p_{i\bar{c}}y_{ic}+\beta \sum_{i=1}^{N}\;\;p_{ic}y_{i\bar{c}}}$$

where $p_{ic}$ is the probability of pixel i being class c, $y_{ic}$ is the ground truth, α controls the penalty for False Positives (FP), and β controls the penalty for False Negatives (FN). In our implementation, we intentionally set β=0.7 and α=0.3 to severely penalize missed cancer detections. To force the model to focus on challenging, hard-to-segment, we apply a focal parameter γ=0.75:

$$L_{FTL}=\sum_{c}\;(1-TI_{c}{)}^{\gamma }$$

The final optimization objective driving our model is a weighted sum designed to offer optimal gradient stability and hard-example mining:

$$L_{Total}=0.5\cdot L_{BCE}+L_{FTL}$$

### 2.9. Optimization Strategy

(a) Optimizer: Network weights were updated using the AdamW (Adaptive Moment Estimation with Decoupled Weight Decay) optimizer. AdamW was selected over traditional Adam or SGD as it properly decouples L2 regularization from the adaptive gradients, yielding improved generalization over biological variance. Weight decay was set to $10^{-4}$ with a base learning rate of $10^{-4}$.

(b) Learning Rate Scheduling: We employed a ReduceLROnPlateau scheduler. The scheduler monitored the validation IoU metric; if the IoU stalled for 5 continuous epochs, the learning rate was decayed by a factor of 0.5. This allows the model to take large steps early in optimization, scaling down dynamically to navigate sharp, narrow local minima in the complex medical loss terrain toward the end of training.

(c) Cross-Validation: To reduce dependence on a single train-validation split and to estimate performance stability across different image-level subsets.

### 2.10. Evaluation Metrics

Segmentation performance was evaluated by comparing predicted binary masks with ground-truth masks. The primary metric was Intersection over Union. Additional metrics included Dice similarity coefficient, recall/sensitivity and specificity. To rigorously evaluate the segmentation performance, the model’s raw output logits were first transformed into probability maps using a Sigmoid activation function. A strict binarization threshold of 0.5 was applied to generate the final discrete segmentation masks. The core evaluation metrics—Intersection over Union (IoU), Dice Similarity Coefficient (DSC), Sensitivity, and Specificity -were computed using a global micro-averaging strategy at the fold level. Specifically, the true positive, false positive, true negative, and false negative pixels were accumulated globally across all images in the validation fold before computing the final metrics. This methodology prevents evaluation biases arising from arbitrary batch allocations or single-image macro-averaging, which is particularly vulnerable to extreme outliers such as completely tumor-negative control samples. To ensure a fair and consistent evaluation, the final model checkpoint for each fold was selected and saved based strictly on achieving the highest validation IoU during the training phase. All subsequent validation metrics reported for a given fold were extracted simultaneously from this single optimal checkpoint. Finally, the overall model performance (Table 1) is reported as the macro-average across the 5 independent cross-validation folds, with the variance expressed as the sample standard deviation across these 5 macro-scores. No threshold-optimization experiments were performed. The binarization threshold was strictly maintained at 0.50 and applied consistently across all folds to avoid post hoc optimization on the validation data.

## 3. Results

### 3.1. Cross-Validation Performance

To ensure the robustness and generalizability of our proposed model, we evaluated its performance using a rigorous five-fold cross-validation strategy. The dataset was partitioned using standard random K-Fold cross-validation, ensuring that an equal number of images were assigned to each fold without artificial stratification, and the model was trained independently on each fold. The final performance metrics are reported as the mean and standard deviation across all five folds, demonstrating high consistency regardless of the specific data split. The evaluation metrics achieved across the cross-validation folds are presented in Table 1. The model achieved a high average Dice Similarity Coefficient (DSC) of 0.8507 ± 0.0182, indicating substantial overlap between the predicted segmentations and the ground truth annotations. Furthermore, the model achieved a mean pixel-level sensitivity of 0.9559 ± 0.0266 and a mean pixel-level background specificity of 0.9956 ± 0.0003. Because the validation dataset did not include entirely tumor-negative images, specificity reflects background-pixel classification within tumor-positive CT slices and should not be interpreted as image-level specificity for excluding colorectal cancer. The narrow standard deviations across all metrics confirm that the model’s performance is stable and not reliant on a specific subset of the training data.

### 3.2. Training Dynamics

The training dynamics of the model were closely monitored to ensure stable convergence and to prevent overfitting. During training, a discriminative learning rate strategy was employed, applying a lower learning rate to the pretrained ResNet50 encoder to preserve the generalized ImageNet features, while allowing the randomly initialized decoder to learn at a standard rate. Training was performed for 25 epochs. Both training and validation losses generally decreased during the initial epochs and showed a tendency toward stabilization during the later stages of the 25-epoch training schedule. Model checkpoints were saved whenever a new best validation performance was achieved. The parallel trajectories of the training and validation loss curves, along with the steady increase in the validation metrics, indicate that the model generalized well to unseen data without exhibiting signs of severe overfitting.

### 3.3. Qualitative Segmentation Examples

A qualitative assessment of the model’s predictions further validates its strong quantitative performance. Visual inspection of the generated segmentation masks reveals a high degree of agreement with the ground truth annotations provided by medical experts.

**Figure 4:**
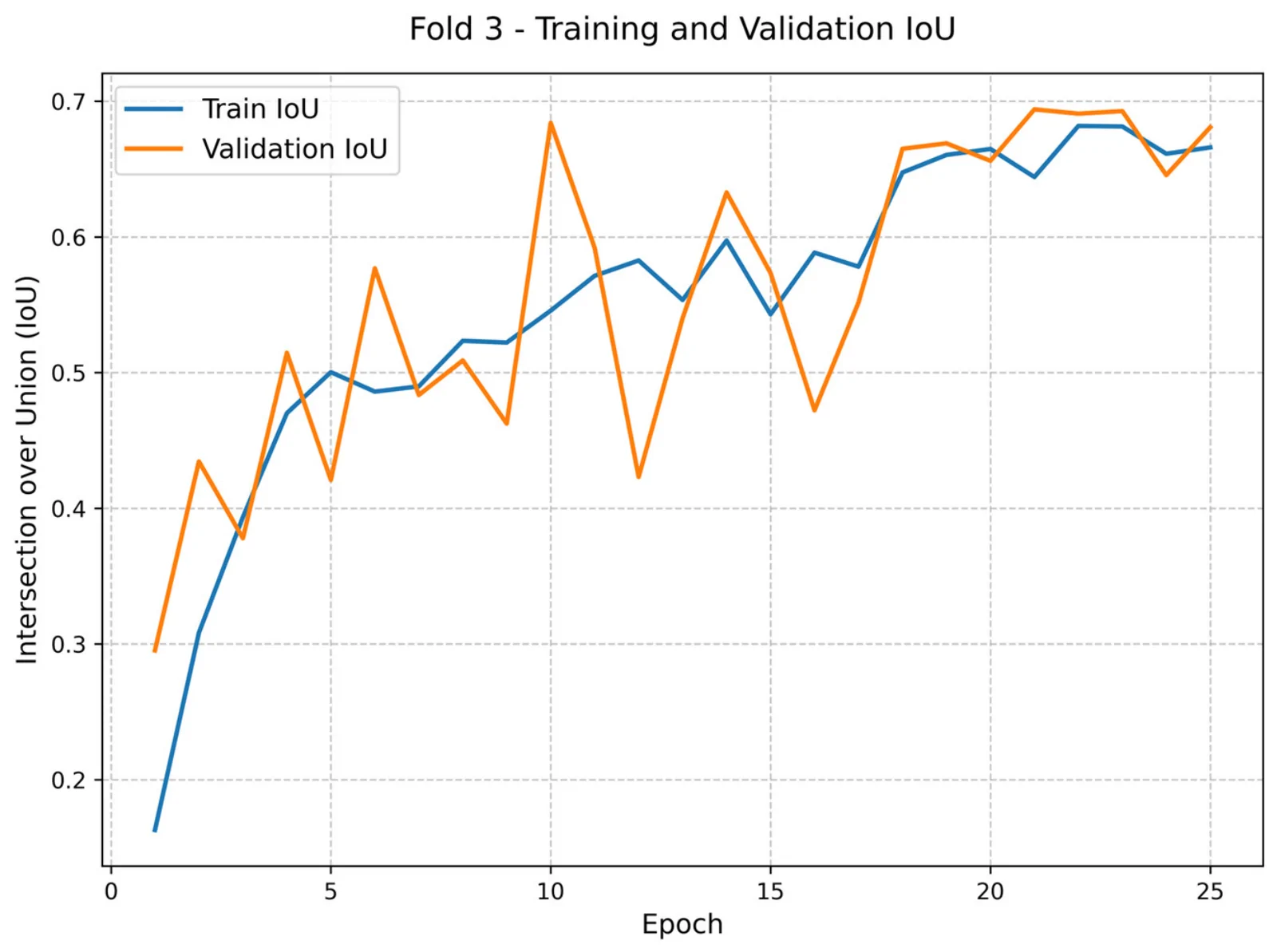
Intersection over Union evolution during 25 epochs for Fold 3. This fold represents the lower bound of the model’s performance variability, yet still demonstrates stable convergence without severe overfitting.

**Figure 5:**
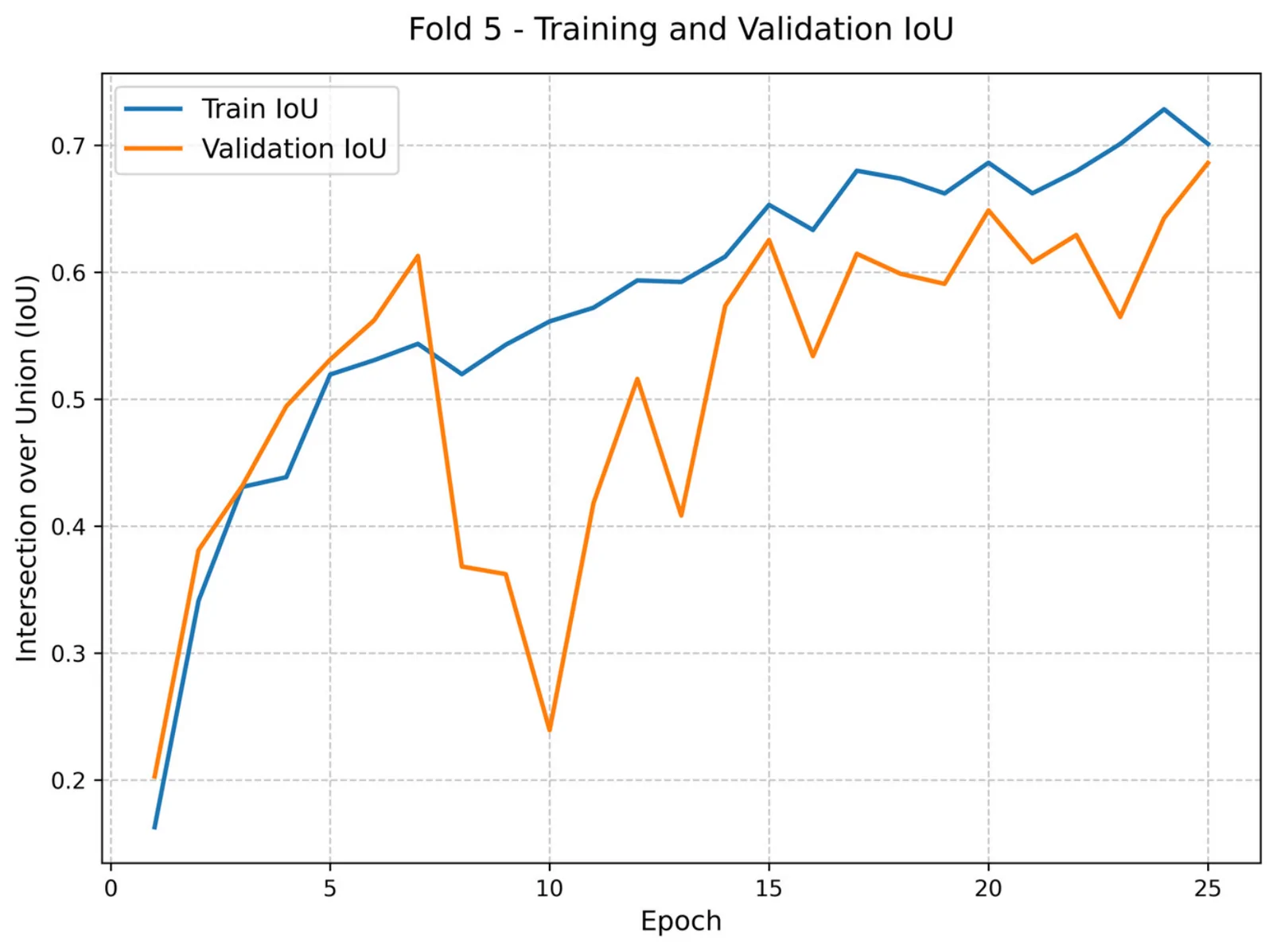
Intersection over Union evolution during 25 epochs for Fold 5. This fold achieved the highest segmentation efficacy, demonstrating rapid early convergence and high validation stability.

**Figure 6:**
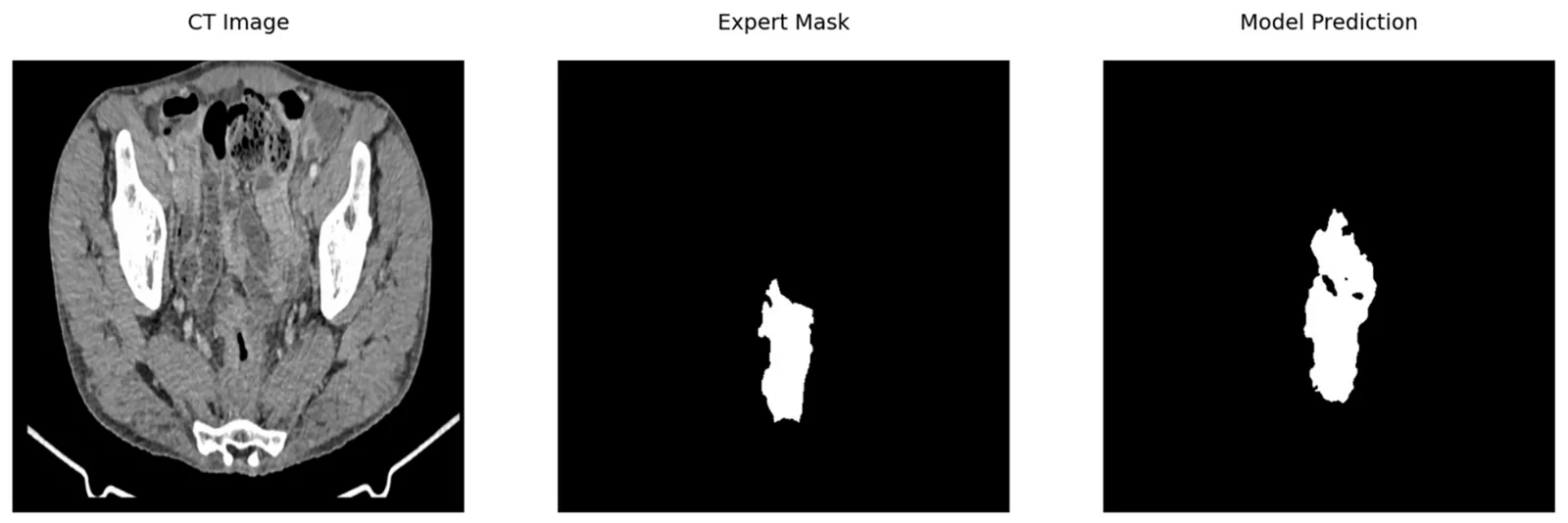
Sample prediction from the validation dataset (I). The model successfully delineates the complex morphological boundaries of the colorectal tumor.

**Figure 7:**
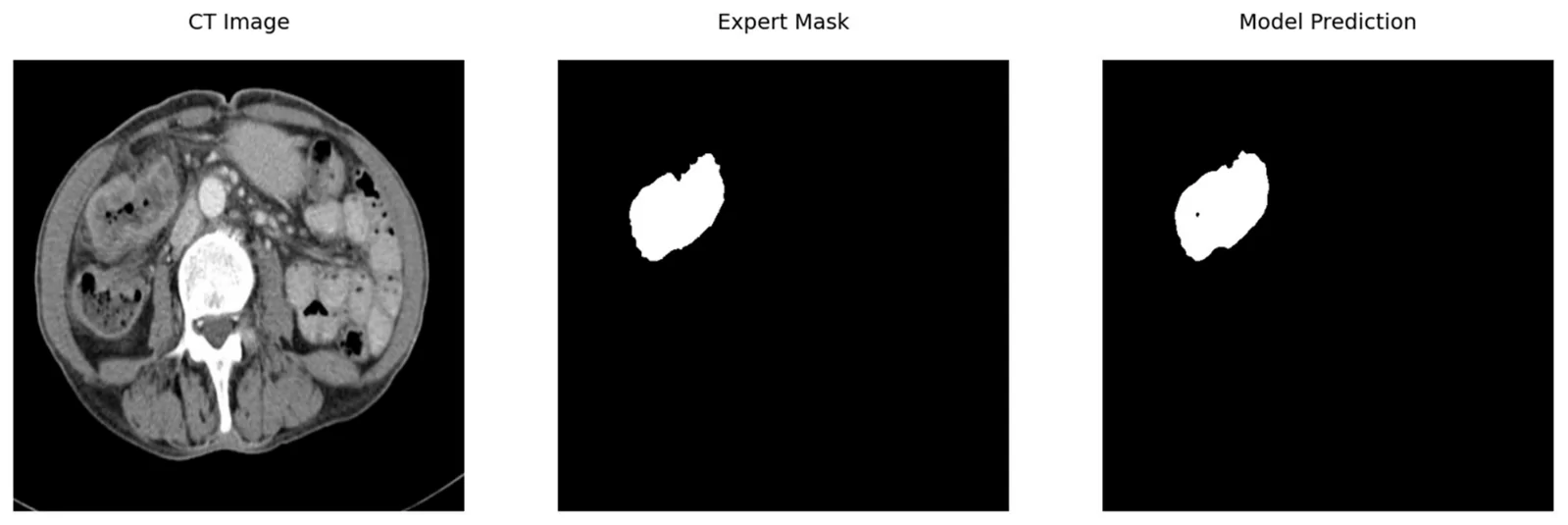
Sample prediction from the validation dataset (II). The predicted segmentation demonstrates good spatial agreement with the reference mask despite the heterogeneous appearance of the surrounding tissues.

As shown in Figure 4, Figure 5, Figure 6 and Figure 7, the model successfully delineates complex tumor boundaries, even in cases with challenging contrast or irregular anatomical structures. The attention mechanisms within the architecture allow the model to focus on the clinically relevant regions of the colon while suppressing background noise. Minor discrepancies between the predictions and ground truth are generally confined to the extreme peripheries of the tumor margins, which inherently suffer from a degree of ambiguity in manual annotations.

### 3.4. Absence of Evaluation on Entirely Tumor-Negative Images

The final dataset did not include entirely tumor-negative CT images with empty ground-truth masks. Consequently, the present study could not evaluate image-level false-positive behavior, the proportion of negative images with any predicted tumor component, or the number and area of false-positive components per negative image. The reported specificity represents pixel-level background specificity within tumor-positive CT slices and should not be interpreted as clinical specificity for ruling out colorectal cancer. Evaluation on an independent cohort of entirely tumor-negative CT examinations will be required in future studies.

To assess the clinical utility of the model, we compare the segmentation masks with expert annotations using metrics that address the significant class imbalance characteristic of CT imaging. Standard pixel-wise accuracy often produces misleadingly high scores due to the predominance of background (non-tumor) pixels, yet it does not adequately reflect the precision of lesion boundaries. Therefore, we prioritize Intersection over Union (IoU) given by Equation (1), as this metric specifically evaluates the spatial overlap between predicted and ground truth regions, offering a more rigorous assessment of segmentation fidelity. The proposed model was trained on a server with Xeon^®^ Platinum 8480+, 256 GB RAM and NVIDIA^®^ H200 GPU. All computations were performed in Python (version 3.14.3) and PyTorch (version 2.11.0). The model was trained for 25 epochs with a batch size of 12. These were obtained by saving the model state every time a new best state was obtained for the model. Figure 4 and Figure 5 illustrate the evolution of the Intersection over Union (IoU) metric over 25 epochs for folds 3 and 5. These particular folds were chosen as they represent the least and most effective performance, respectively. Sample predictions of the proposed model are presented in Figure 6 and Figure 7.

Intersection over Union (IoU/Jaccard Index):

(1) $$IoU=\frac{\left|Y\cap \hat{Y}\right|}{\left|Y\cup \hat{Y}\right|}=\frac{TP}{TP+FP+FN}$$

### 3.5. Ablation Study and Baseline Comparisons

To empirically evaluate the architectural contributions of the deep encoder and the attention mechanism, an ablation study was performed. The proposed ResNet50-Attention-UNet was compared against three baseline architectures: a standard U-Net, an Attention U-Net, and a ResNet50-UNet without attention gates. The results obtained are presented in Table 2. Compared with the standard U-Net, the ResNet50 encoder substantially improved segmentation overlap. Adding attention gates to the ResNet50–U-Net further increased Dice, IoU, and sensitivity, while pixel-level background specificity remained comparably high. These findings suggest that the attention mechanism primarily improved tumor-region preservation and spatial overlap rather than background specificity.

### 3.6. Computation Efficiency

To evaluate the feasibility of deploying the proposed ResNet50-Attention-UNet in real-world clinical environments, we assessed both the training and inference computational requirements. During the training phase, the model required a peak GPU memory allocation of 13,377.70 MB, easily accommodating standard high-performance research hardware. More importantly, during the evaluation phase, the model demonstrated high efficiency for clinical deployment. Processing a single CT slice required an average inference time of just 6.70 ms (on the same training server), enabling fast segmentation of CT slices. The relatively low inference memory requirement and short per-slice processing time indicate computational efficiency on the evaluated hardware. However, deployment performance on standard clinical workstations and complete DICOM volumes requires separate validation. All of the computation tests were run on the same server used for training.

## 4. Discussion

### 4.1. Principal Findings

In this study, we developed and evaluated an attention-enhanced ResNet50-U-Net architecture for automated colorectal tumor segmentation on contrast-enhanced abdomino-pelvic CT images. The proposed model was designed to combine the hierarchical feature extraction capacity of a pre-trained ResNet50 encoder with the spatial localization ability of an attention-guided U-Net decoder. Using five-fold image-level cross-validation, the model achieved a mean validation Intersection over Union (IoU) of 0.7406, indicating moderate-to-good segmentation performance across validation subsets.

The results suggest that attention-enhanced encoder–decoder architectures may support automated colorectal tumor delineation in routine CT images, particularly in the context of heterogeneous tumor morphology and complex abdominal anatomy. Qualitative examples showed broad spatial agreement between predicted masks and expert-derived annotations, although minor boundary differences and small false-positive components were observed. Therefore, the findings should be interpreted as preliminary technical evidence supporting the feasibility of colorectal tumor segmentation on CT, rather than as proof of clinical deployment readiness.

### 4.2. Interpretation of Segmentation Performance

Automated colorectal tumor segmentation on CT is technically challenging because tumor conspicuity depends on multiple factors, including lesion size, bowel distension, luminal content, contrast enhancement, reconstruction characteristics, pericolic fat planes, and the presence or absence of secondary inflammatory changes. In addition, colorectal tumors often have irregular margins and variable enhancement, and they may be difficult to separate from collapsed bowel loops, fecal material, adjacent vessels, mesenteric fat, pelvic structures, or inflammatory changes.

In this context, a mean IoU of 0.7406 represents a reasonable preliminary result for a heterogeneous image-level dataset. The relatively narrow variation across folds suggests that the model learned reproducible image features within the available cross-validation framework. However, this value should not be overinterpreted. The present analysis was performed at image level, and complete patient-level identifiers were not available for all public-source images. As a result, the reported performance reflects preliminary image-level segmentation behavior rather than definitive patient-level generalization.

The qualitative predictions further support this interpretation. The model was able to localize the main tumor region and generate masks that were broadly consistent with expert annotations. Nevertheless, segmentation boundaries were not always identical to the reference masks, and isolated false-positive pixels may occur. Such behavior is expected in raw deep learning segmentation outputs, particularly in complex abdominal CT images. Future post-processing strategies, such as connected-component filtering or morphological refinement, may reduce small isolated false-positive components, but these methods should be evaluated systematically before being incorporated into a clinical pipeline.

### 4.3. Comparison with Previous Studies

The recent literature suggests that automated colorectal tumor segmentation and detection remain heterogeneous tasks, with reported performance depending strongly on imaging modality, acquisition protocol, annotation strategy, cohort size, validation design, and whether the algorithm targets tumor segmentation, lesion localization, or broader colorectal anatomy. Consequently, direct comparison between studies must be made cautiously. A comparison with existing works is presented in Table 3.

Routine contrast-enhanced abdominal CT appears to be more challenging than highly standardized imaging settings. Recent work has shown that robust colorectal tumor segmentation on ordinary CT remains difficult, even with modern deep learning approaches [18]. Chen et al. developed an nnU-Net-based tumor segmentation model in a multicenter cohort of 2180 patients, divided into training (n = 1159), validation (n = 289), and independent external test (n = 732) cohorts. In the independent external test cohort, the arterial-phase segmentation model achieved a Dice coefficient of 0.71 [19]. These findings support the feasibility of automated colorectal tumor segmentation while also emphasizing the importance of patient-level external validation.

Yao et al. developed DeepCRC-SL, a colorectal coordinate-driven method for automated segmentation of colorectal cancer and the colorectum on conventional contrast-enhanced CT scans. The method was evaluated using five-fold cross-validation on 812 colorectal cancer cases, including 227 labeled and 585 unlabeled cases, and achieved Dice coefficients of 0.669 for colorectal cancer and 0.892 for the colorectum [20]. Although these findings support the value of topology-aware and self-learning approaches, differences in dataset composition, model architecture, and validation design limit direct comparison with the present image-level study.

Other studies have explored architectural refinements for colorectal CT segmentation. Pei et al. reported relative improvements in Dice performance over U-Net and CE-Net, supporting the potential value of attention mechanisms in colorectal CT segmentation [21]. Similarly, the MDCC-Net study reported a Dice coefficient of 83.57 and highlighted the relevance of multiscale U-Net-based designs for this task [22]. Residual feature extraction has also shown promise; Akilandeswari et al. reported high segmentation scores using a residual CNN approach, including an average Dice score of 91.57, sensitivity of 98.28, and specificity of 98.68, suggesting that residual encoders may be effective in curated CT datasets [23]. These studies support the rationale for combining residual feature extraction with attention-guided decoding, although differences in dataset composition, preprocessing, annotation, and validation strategy limit direct quantitative comparison.

The broader literature on colorectal cancer detection also provides relevant context. Kim et al. evaluated automatic colorectal cancer detection on routine contrast-enhanced abdomino-pelvic CT scans, emphasizing the relevance of AI in non-standardized clinical imaging environments and reporting AUC values exceeding 0.80 in internal and external testing [24]. Yao et al. similarly investigated opportunistic colorectal cancer detection on standard contrast-enhanced CT without bowel preparation and reported high AUC values across multiple cohorts, demonstrating that sufficiently diverse training data may support generalization across variable CT protocols [25]. However, these studies primarily address lesion detection rather than pixel-level tumor segmentation. Therefore, they are complementary to the present work but not directly equivalent.

Studies based on CT colonography also demonstrate the importance of imaging protocol standardization. Endo et al. developed a deep learning CAD system for CT colonography using standardized bowel preparation, luminal distension, and supine/prone acquisitions. Although the model achieved promising internal performance, external validation was less robust, especially for smaller lesions, highlighting the difficulty of cross-institutional generalization even in controlled imaging settings [26]. This observation is relevant to the present study because routine abdomino-pelvic CT is even more variable than CT colonography.

Other imaging-based AI studies in colorectal cancer further reinforce the importance of methodological standardization. Chen et al. showed, in a systematic review and meta-analysis, that AI models for imaging-based prediction of distant metastasis in colorectal cancer can achieve promising diagnostic performance, but the included studies were limited by substantial heterogeneity in imaging modalities, reference standards, and validation methods [27]. Li et al. demonstrated that AI-assisted iterative reconstruction can improve CT image quality in colorectal cancer assessment, emphasizing that reconstruction algorithms and image appearance may influence both human interpretation and AI model behavior [28]. Cao et al. showed that U-Net-based segmentation can achieve high precision for CT-based body composition analysis in colorectal cancer patients, supporting the broader feasibility of automated CT segmentation tasks [29]. Ameyaw et al. also highlighted persistent barriers to clinical translation, including inconsistent acquisition protocols, difficulty with small or flat lesions, and the limited availability of externally validated multicenter studies [30]. Finally, Wu et al. demonstrated that routine CT contains prognostically relevant information for colorectal cancer recurrence prediction when radiomic and clinical features are integrated, supporting the broader value of computational analysis beyond visual interpretation alone [31].

Taken together, the existing literature supports the relevance of the present work, but also emphasizes the need for cautious interpretation. The current study contributes preliminary evidence for attention-enhanced ResNet-U-Net segmentation of colorectal tumor regions on CT, but future patient-level and external validation will be required to establish robust generalizability.

### 4.4. Technical Relevance of Attention and ResNet Encoder

The proposed architecture was designed to address two major technical challenges in colorectal tumor segmentation: complex feature extraction and suppression of irrelevant background structures. The ResNet50 encoder provides deep hierarchical feature representations and may reduce the risk of unstable training compared with training a deep network from scratch on a limited tumor-positive dataset. Residual connections facilitate gradient propagation and support the extraction of more complex imaging features, which is relevant for colorectal tumors with variable texture, enhancement, and morphology.

The attention-guided decoder was incorporated to refine skip connections and reduce the influence of irrelevant activations from adjacent abdominal structures. In colorectal CT imaging, standard U-Net skip connections may transmit both useful spatial information and distracting background features, including bowel contents, vessels, mesenteric fat, pelvic bones, and adjacent soft tissues. Attention gates may help suppress such non-tumor activations while preserving tumor-related features, thereby improving localization of the region of interest.

The use of a composite Binary Cross-Entropy and Focal Tversky Loss was intended to address the severe foreground–background imbalance typical of tumor segmentation. Colorectal tumor pixels usually occupy a small proportion of the axial CT image, while background pixels dominate the training signal. By penalizing false-negative predictions more strongly, Focal Tversky Loss may encourage the network to preserve tumor regions that would otherwise be under-segmented. This is particularly relevant in oncologic imaging, where missing tumor tissue may be more clinically consequential than generating small false-positive components. The architectural ablation study demonstrated incremental improvements associated with the ResNet50 encoder and attention gates. However, the individual contribution of the composite loss function was not separately evaluated, and direct comparison with self-configuring frameworks such as nnU-Net was not performed.

### 4.5. Clinical Relevance

Accurate colorectal tumor segmentation on CT may have several potential downstream applications. Automated delineation could support quantitative tumor assessment, tumor volume estimation, radiomics feature extraction, treatment response evaluation, surgical planning, and radiotherapy pre-treatment workflows. In high-volume radiology departments, AI-assisted segmentation may also help reduce the manual workload associated with lesion contouring and provide more standardized tumor measurements.

However, the present study should not be interpreted as a clinical diagnostic tool or as a replacement for colonoscopy, histopathological diagnosis, or expert radiological interpretation. The model was evaluated as a technical segmentation approach on selected axial CT images, not as a full-volume detection system for screening or opportunistic diagnosis. Clinical implementation would require reliable patient-level performance, external validation, integration into a DICOM-based workflow, prospective testing, and evaluation of reader interaction, false positives, and failure modes.

Therefore, the most appropriate interpretation is that this study represents an early technical step toward AI-assisted colorectal tumor delineation on CT. Its potential clinical value lies in supporting future workflows for quantitative imaging and decision support rather than in immediate autonomous diagnosis.

### 4.6. Limitations

This study has several important limitations. First, as a principal methodological constraint, validation was performed strictly at the image level because complete patient-level identifiers were not available for the anonymized public datasets. Consequently, adjacent or related slices from the same patient could not be systematically excluded from crossing the train-validation boundary. This introduces a risk of data leakage and optimistic bias, limiting our conclusions regarding patient-level generalizability. Therefore, large-scale, multi-institutional external validation on strictly patient-stratified cohorts remains an absolute prerequisite before this tool can be considered for clinical deployment.

Second, the dataset was based on exported PNG images rather than original DICOM volumes. As a result, voxel-level Hounsfield Unit information, slice thickness, reconstruction metadata, and full volumetric context were not consistently available during training. This limits reproducibility and may reduce the model’s ability to learn scanner-independent quantitative CT features.

Third, the current model operates on axial images. Although this approach is technically simpler and allows direct slice-level segmentation, it does not capture the continuous craniocaudal extent of colorectal tumors or the three-dimensional relationship between tumor, bowel wall, mesentery, vessels, and adjacent organs. A 3D or hybrid 2.5D/3D approach may better capture volumetric tumor morphology.

Fourth, the study lacks an independent external test set. Although images were obtained from internal and public sources, the model was not evaluated on a fully held-out external cohort with patient-level separation. External validation across multiple institutions, CT vendors, acquisition protocols, and reconstruction algorithms is necessary before clinical translation.

Finally, a limitation involves the composition of the dataset, which exclusively comprises tumor-positive images. Because entirely tumor-negative control scans were not included, the clinical specificity for decisively ruling out pathology in a general screening population cannot be definitively established. The reported specificity is therefore confined to pixel-level background suppression within positive slices. Future large-scale cohort studies incorporating healthy control scans are required to thoroughly validate the model’s false-positive behavior at the examination level.

### 4.7. Future Work

Future studies should focus on patient-level and external validation using larger multi-institutional datasets with complete DICOM metadata. A fully DICOM-based workflow would preserve Hounsfield Unit information, acquisition parameters, slice spacing, and volumetric context, improving reproducibility and clinical relevance. In addition, future models should incorporate 3D or hybrid 2.5D/3D architectures to better capture inter-slice tumor continuity and anatomical relationships. To facilitate external validation, the source code, trained model weights, and preprocessing pipeline are available to researchers upon reasonable academic request, and the authors are open to analyzing eligible external CT datasets through collaborative studies, subject to appropriate ethical and data-governance approvals.

Finally, clinical translation will require prospective validation, assessment of inference time, integration with radiology workflows, explainability analysis, and evaluation of how radiologists interact with model outputs. Only after such validation can attention-enhanced segmentation systems be considered for routine decision-support applications in colorectal cancer imaging.

## 5. Conclusions

This study developed and evaluated an attention-enhanced ResNet50-U-Net architecture for automated colorectal tumor segmentation on contrast-enhanced abdomino-pelvic CT images. The model achieved a mean validation IoU of 0.7406 across five-fold image-level cross-validation, suggesting that attention-enhanced encoder–decoder architectures may support colorectal tumor delineation in routine CT images.

The proposed approach represents a preliminary technical step toward AI-assisted colorectal tumor segmentation rather than a clinically validated diagnostic system. Its potential value lies in future applications such as quantitative tumor assessment, radiomics, treatment planning, and workflow support. However, further validation on larger, patient-level, multi-institutional DICOM-based datasets is required before clinical translation.

## Figures and Tables

**Table 1:** Five-fold Cross-Validation Results.

Metric	Fold 1	Fold 2	Fold 3	Fold 4	Fold 5	Mean ± Standard Deviation 95% CI
Dice Similarity Coefficient (DSC)	0.8377	0.8458	0.8314	0.8643	0.8745	0.8507 ± 0.0182 [0.8282, 0.8732]
Intersection over Union (IoU)	0.7207	0.7328	0.7115	0.7610	0.7770	0.7406 ± 0.0276 [0.7063, 0.7749]
Pixel-level—Sensitivity (Recall)	0.9796	0.9433	0.9201	0.9524	0.9843	0.9559 ± 0.0266 [0.9229, 0.9889]
Pixel-level—Specificity	0.9951	0.9960	0.9955	0.9958	0.9958	0.9956 ± 0.0003 [0.9952, 0.9960]

**Table 2:** Ablation Study and Baseline Comparisons.

Architecture	Dice (%)	IoU (%)	Sensitivity (%)	Specificity (%)
U-Net (base)	71.49 ± 1.75	56.41 ± 2.24	80.42 ± 6.66	99.33 ± 0.28
Attention U-Net	72.45 ± 1.36	57.59 ± 1.52	84.85 ± 6.36	99.26 ± 0.16
ResNet50-U-Net	81.81 ± 2.79	69.98 ± 3.66	87.71 ± 5.67	99.61 ± 0.14
**ResNet50-Attention-U-Net (proposed)**	**85.07 ± 1.82**	**74.06 ± 2.76**	**95.59 ± 2.66**	**99.56 ± 0.03**

**Table 3:** Comparison with other studies.

Study	Patients	Images/Scans	IoU	Dice Coefficient
Yao et al. [18]—DeepCRC	107 cases	107 3D CT scans	Not reported	CRC: 0.646 ± 0.275; colorectum: 0.862 ± 0.058
Chen et al. [19]	2180 patients	1159 training, 289 validation, and 732 independent external test	Not reported	External test, arterial phase: 0.71
Yao et al. [20]	812 cases total: 227 labeled and 585 unlabeled	812 3D CT volumes	Not reported	CRC: 0.669; colorectum: 0.892
Pei et al. [21]	158 patients	1131 2D images	0.5782	0.7089
Zheng et al. [22]—MRI cases	200 patients	1102 tumor-containing 2D slices	Not reported	0.8357
Proposed method	70 institutional images obtained from 25 patients and 423 images from publicly available colorectal cancer imaging resources		0.7406	0.8507

## Data Availability

The raw data supporting the conclusions of this article will be made available by the authors on request.
